# Human B-1 cells are important contributors to the naturally-occurring IgM pool against the tumor-associated ganglioside Neu5GcGM3

**DOI:** 10.3389/fimmu.2022.1061651

**Published:** 2022-11-30

**Authors:** Nely Rodriguez-Zhurbenko, Tam D. Quach, Thomas L. Rothstein, Ana M. Hernandez

**Affiliations:** ^1^ Immunology and Immunotherapy Division, Center of Molecular Immunology, Havana, Cuba; ^2^ Institute of Molecular Medicine, The Feinstein Institutes for Medical Research, Manhasset, NY, United States; ^3^ Center for Immunobiology and Department of Investigative Medicine, Western Michigan University Homer Stryker M.D. School of Medicine, Michigan, MI, United States; ^4^ Biochemistry Department, Faculty of Biology, Havana University, Havana, Cuba

**Keywords:** human B-1 cells, natural antibodies, gangliosides, tumor, Neu5GcGM3

## Abstract

Only few studies have described the anti-tumor properties of natural antibodies (NAbs). In particular, natural IgM have been linked to cancer immunosurveillance due to its preferential binding to tumor-specific glycolipids and carbohydrate structures. Neu5GcGM3 ganglioside is a sialic acid–containing glycosphingolipid that has been considered an attractive target for cancer immunotherapy, since it is not naturally expressed in healthy human tissues and it is overexpressed in several tumors. Screening of immortalized mouse peritoneal-derived hybridomas showed that peritoneal B-1 cells contain anti-Neu5GcGM3 antibodies on its repertoire, establishing a link between B-1 cells, NAbs and anti-tumor immunity. Previously, we described the existence of naturally-occurring anti-Neu5GcGM3 antibodies with anti-tumor properties in healthy young humans. Interestingly, anti-Neu5GcGM3 antibodies level decreases with age and is almost absent in non-small cell lung cancer patients. Although anti-Neu5GcGM3 antibodies may be clinically relevant, the identity of the human B cells participating in this anti-tumor antibody response is unknown. In this work, we found an increased percentage of circulating human B-1 cells in healthy individuals with anti-Neu5GcGM3 IgM antibodies. Furthermore, anti-Neu5GcGM3 IgMs were generated predominantly by human B-1 cells and the antibodies secreted by these B-1 lymphocytes also recognized Neu5GcGM3-positive tumor cells. These data suggest a protective role for human B-1 cells against malignant transformation through the production of NAbs reactive to tumor-specific antigens such as Neu5GcGM3 ganglioside.

## Introduction

NAbs are spontaneously and constitutively secreted in the absence of exogenous antigenic stimulation. These immunoglobulins recognize antigens highly conserved throughout evolution, including phospholipids, oxidized lipids, glycolipids, glycoproteins and carbohydrates (1). Such reactivity allows NAbs to play an important role in the defense against invading pathogens, the maintenance of tissue homeostasis, and the protection against atherosclerosis and malignant transformation (1, 2).

In humans, many naturally-occurring antibodies recognize glycan epitopes on both glycoproteins and glycolipids from different tissues. Among the carbohydrate structures recognized by these anti-glycan antibodies are the blood group antigens A and B, the xenoantigen Galα1-3Galβ1-4GlcNAc, Forssman glycolipid antigen, Hanganutziu–Deicher antigen, and gangliosides (3–5).

Gangliosides are sialic acid–containing glycosphingolipids widely expressed in the plasma membrane of essentially all vertebrate cells (6). The ganglioside composition of cell membranes suffers significant changes during malignant transformation. Neu5GcGM3 is among the gangliosides that more drastically change during tumorigenesis, being absent in normal human tissues but present in a wide variety of human tumors (7).

Previously, we demonstrated the existence of naturally-occurring anti-Neu5GcGM3 antibodies in the sera of healthy individuals. These antibodies recognize and eliminate tumor cells bearing Neu5GcGM3. The level of these naturally-occurring antibodies and their anti-tumor cytotoxic capacity significantly decrease in elderly people, when cancer risk is higher. More interestingly, non-small cell lung cancer (NSCLC) patients lack or have very low levels of anti-Neu5GcGM3 antibodies (3), reinforcing the idea of a protective anti-tumor role for these molecules. One possible explanation for the decrease in anti-Neu5GcGM3 antibody levels with increasing age or in NSCLC patients involves an impairment in the frequency or function of the B-cell population responsible of producing these antibodies.

NAbs are predominantly generated by a unique subpopulation of B cells termed B-1 cells. The origin and function of this B-cell population have been most extensively studied in mice, where B-1 cells are identified as CD19^high^B220^low^CD23^-^CD43^+^IgM^high^IgD^low^ (1, 8). The expression of CD5 further divides mouse B-1 cells in two distinct populations: B-1a (CD5+) and B-1b (CD5-) cells. In humans, B-1 cells have been identified as CD19+CD20+CD38^low/int^CD27+CD43+ based on the fact that B cells with this phenotype fulfill key functional criteria characteristic of mouse B-1 cells (9, 10). This phenotypic profile has been used by an increasing number of investigators in translational studies of specific disease states (11–15). Recently, Suo et al. identified, characterized and functionally validated the properties of human prenatal B-1 cells. The authors demonstrated that prenatal B-1 cells with the same phenotypic markers described by Griffin et al. (9) share distinctive characteristics with mouse B-1 cells including self-renewal, high IgM and low IgD expression, emergence in early stages of development, low levels of N/P-additions, tonic BCR signaling, and spontaneous antibody secretion (16).

B-1 cells take part in host immunosurveillance against cancer. A group of recent reports described a protective role for natural, tumor-reactive IgM secreted by B-1a cells in a mouse model of peritoneal carcinomatosis (17, 18). Also, monoclonal IgM antibodies derived from B-1 cells expressing PD-L2 bind and induce cell death of a colon cancer cell line (19). Rawat et al. suggested that NAbs play an important role in the elimination of neoantigen-expressing cells, and consequently in anti-tumor immunity (20). Our previous studies demonstrated that peritoneal B-1 cells contain on their repertoire anti-Neu5GcGM3 antibodies able to bind and eliminate Neu5GcGM3-positive tumor cells (21). However, the B-cell population responsible for anti-Neu5GcGM3 in humans has not been identified. Here, we examined the ability of three human B-cell subpopulations to secrete IgM antibodies reactive to the tumor-specific antigen Neu5GcGM3. A primary role for human B-1 cells in anti-Neu5GcGM3 antibodies secretion was stablished. These findings confirm the protective function of B-1 cells against tumors through the production of antibodies reactive to tumor-specific antigens such as Neu5GcGM3 ganglioside.

## Materials and methods

### Gangliosides and tumor cell lines

Gangliosides Neu5AcGM3 and Neu5GcGM3 were provided by Dr. L.E. Fernandez (Center of Molecular Immunology, Cuba). P3-X63-Ag8.653 murine myeloma cell line (X63) was obtained from the American Tissue Type Culture Collection. Cells were grown in DMEM (Gibco) supplemented with 10% fetal calf serum [(FCS), Invitrogen] and maintained at 37°C with 5% CO_2_.

### Human samples

Peripheral blood samples were obtained by venipuncture from 78 healthy individuals. Among the participants, there were 48 females and 30 males. The age range of the studied population was 20 to 88 years with an average age of 49.42 and a standard deviation of 18.73. Participants were selected on the basis of good health status and no reports of infection/immunization within 4 weeks of blood draws. All donors provided written informed consent to participate in the study as approved by the North Shore-LIJ Health System Institutional Review Board.

### Plasma and cell isolation

Plasma samples were obtained by centrifugation at 100 × g for 10 min. Samples were stored at -80 °C until their use. Peripheral blood mononuclear cells (PBMCs) were isolated by density gradient centrifugation using lymphocyte separation medium (Cellgro). Cells were washed with phosphate-buffered saline (PBS) containing 1 mM EDTA, and resuspended in either cell-sorting buffer [0.5% bovine serum albumin (BSA) in PBS] or flow-cytometry staining buffer (2.5% FCS, 1 mM EDTA in PBS).

### B-cell analysis

For B-cell population analysis, single-cell suspensions (1-2x10^6^/sample) in flow-cytometry staining buffer were blocked with normal mouse serum (Life Technologies) and stained with anti-CD19-allophycocyanin-Alexa Fluor 700 (J3-119), anti-CD20-Pacific Blue (B9E9), anti-CD3-ECD (UCHT1), anti-CD4-ECD (SFCI12T4D11), anti-CD7-ECD (8H8.1), anti-CD27-allophycocyanin (1A4CD27), anti-CD38-PerCP-Cy5.5 (LS198-4-3), anti-CD43-APC-AF750 (DTF1), and Live/Dead Fixable Aqua Dead Cell Stain Kit (Life Techonologies) on ice for 20 min. Fluorescence Minus One (FMO) controls were employed for the analysis. The gating strategy used to identify the different B cell subsets is represented in **Supplementary Figure 1**. Immunofluorescently stained cells were examined on a Beckman-Coulter Gallios cytometer and subsequently analyzed with FlowJo 10.0 software (BD Bioscience).

### Cell sorting

PBMCs were treated with 2.5% NMS in PBS and stained with an antibody mixture consisting of anti–CD19-APC-AF700, anti–CD27-APC, anti–CD43-FITC, anti–CD38-PerCP-Cy5.5, anti–CD20-Pacific Blue, anti–CD3-ECD, anti–CD4-ECD, and anti–CD7-ECD in cell-sorting buffer, and then stained with Live/Dead Fixable Aqua Dead Cell Stain Kit. After washing, cells were resuspended in cell-sorting buffer and sort-purified on an Influx instrument (BD). Mature/naive (CD19+CD20+CD38^lo/int^CD27-CD43-), memory (CD19+CD20+CD38^lo/int^CD27+CD43-) and B-1 (CD19+CD20+CD38^lo/int^CD27+CD43+) were collected from the gated live CD3/4/7 negative population. The average purity of each B cell population was above 90%. Antibodies were purchased from Beckman Coulter.

### B-cell culture

Cells (5-10x10^3^) of each sorted population were cultured in 96-well plates with 200 μL of complete media: RPMI 1640 media (Gibco) supplemented with 10% FCS, 10 mM HEPES, 1 mM non-essential amino acids, 2 mM L-glutamine, 50 µg/mL penicillin/streptomycin and 1 mM sodium pyruvate (Gibco). All B cells were stimulated with 1 μg/mL of CpG oligodeoxynucleotide 2006 (*In vivo*Gen), 2.5 μg/mL goat anti-human immunoglobulin A (IgA) IgG IgM (Fab’)2 (Jackson ImmunoResearch Laboratories), and 10 ng/mL of IL-2. Cells were placed in 5% CO_2_ for 7 days at 37°C and cell culture supernatant was collected by centrifugation.

### Total and specific IgM analysis

Quantity of total IgM in cell culture supernatants of activated B cells was measured by ELISA according to the manufacturer’s instruction (Bethyl Laboratories).

Anti-Neu5GcGM3 IgM antibodies present in healthy donors plasma or in B-cell culture supernatants were detected by ELISA as previously described (22). Plasma samples were evaluated at 1:50 dilution. Cell culture supernatants were evaluated at a final concentration of 100 μg/mL total IgM. To consider that a sample had a positive reaction to a ganglioside, values of absorbance had to be ≥0.25 and at least three times the absorbance value obtained by incubating the serum in wells containing no ganglioside (22). Assays were performed in duplicate for each sample. The optical densities (ODs) of the blanks were less than 0.1.

### Binding to tumor cells

X63 tumor cells were blocked in PBS containing 1% FCS for 20 min on ice. B-cell culture supernatants, diluted to a final concentration of 100 μg/mL total IgM, were incubated with 10^5^ X63 cells for 30 min on ice. After washing with cold PBS, cells were incubated with FITC-conjugated goat anti-human IgM (Jackson ImmunoResearch Laboratories) for 30 min on ice. The percentage of positively stained cells was determined in a Gallios cytometer. Data was analyzed using FlowJo 10.0 software. For a B-cell supernatant to be considered positive, the percentage of binding had to be ≥15% and at least two times the percentage obtained by incubating the cells only with FITC-conjugated goat anti-human IgM.

### Statistics

Graphs construction and statistical analysis was carried out with GraphPad Prism 6.0. To examine differences between two or more groups, Mann-Whitney U-test and Dunn’s test were used, respectively. Error bars represent standard error of mean (SEM).

## Results

### Identifying human B cells secreting anti-Neu5GcGM3 antibodies within multiple B-cell subsets

**Figure 1:**
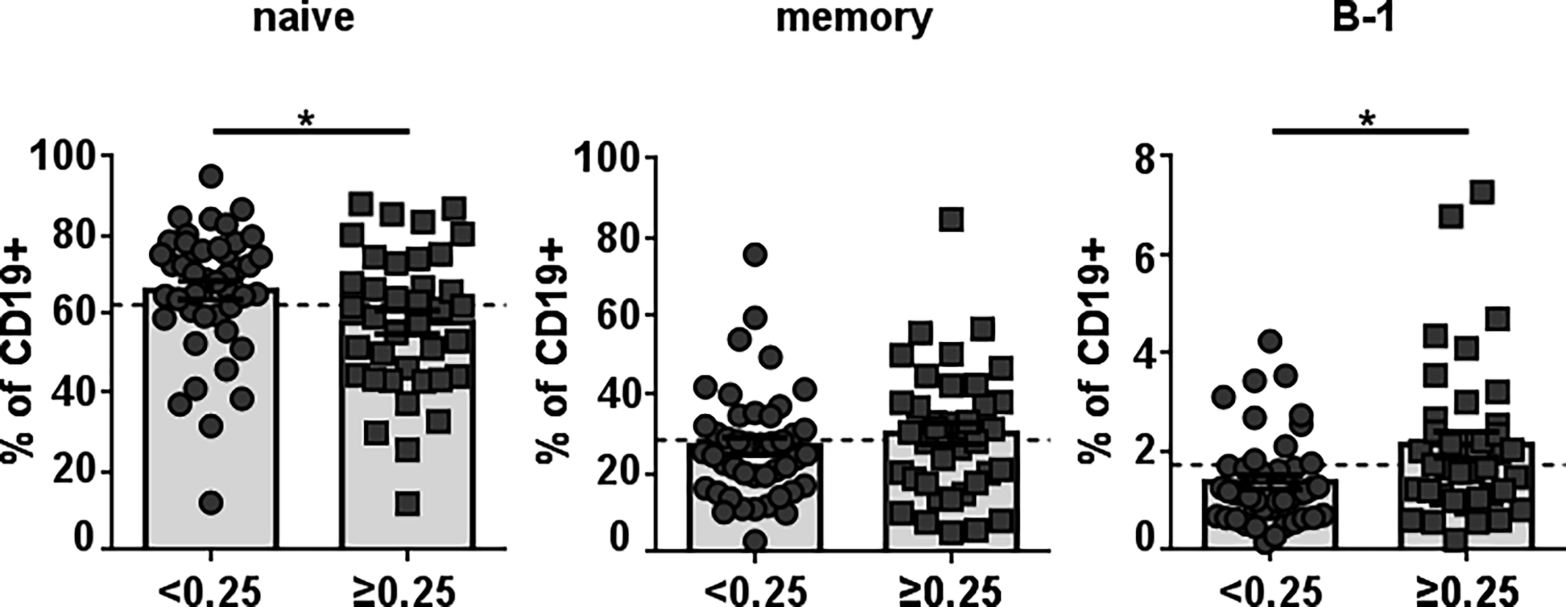
Frequency of different B-cell populations in healthy individuals according to their level of anti-Neu5GcGM3 IgM antibodies. Plasma samples from 78 healthy donors were screened by ELISA for anti-Neu5GcGM3 IgM. Samples diluted 1:50 were assessed on plates coated with Neu5GcGM3 using biotinylated goat anti-human IgM, followed by the addition of an alkaline phosphatase-streptavidin complex. Samples with absorbance values ≥ 0.25 were considered positive to Neu5GcGM3. Percentages of circulating mature/naive (CD19+CD20+CD38^low/int^CD27-CD43-), memory (CD19+CD20+CD38^low/int^CD27+CD43-) and B-1 (CD19+CD20+CD38^low/int^CD27+CD43+) within the CD19+ population in healthy donors without (D.O. < 0.25) or with (D.O. ≥ 0.25) anti-Neu5GcGM3 IgM were analyzed by flow cytometry. *p ≤ 0.05, Mann–Whitney U-test. Each symbol represents an individual healthy donor and the bars represent the means. Dotted lines represent the mean of the total memory, mature/naive or B-1 cell population, respectively.

To identify healthy donors with naturally-occurring antibodies against Neu5GcGM3, we examined anti-Neu5GcGM3 IgM antibodies in 78 plasma samples from healthy humans of different ages. Similar to our previous studies (3, 22) we detected the existence of naturally-occurring anti-Neu5GcGM3 IgM antibodies in 36 healthy humans (46% of the samples). As a first approach to identify the B-cell population responsible for anti-Neu5GcGM3 IgM secretion we measured the frequency of circulating mature/naive (CD19+CD20+CD38^low/int^CD27-CD43-), memory (CD19+CD20+ CD38^low/int^CD27+CD43-) and B-1 (CD19+CD20+CD38^low/int^CD27+CD43+) cells in healthy donors with or without anti-Neu5GcGM3 antibodies. No significant difference was observed in the percentage of memory cells between donors with or without circulating anti-Neu5GcGM3 antibodies. The mature/naive B-cell population was higher in those healthy donors without anti-Neu5GcGM3 IgM antibodies. Interestingly, B-1 cell frequency was significantly higher in individuals that resulted positive for anti-Neu5GcGM3 antibodies (**Figure 1**). These results suggest that human B-1 cell may be involved in anti-Neu5GcGM3 IgM antibody secretion.

**Figure 2:**
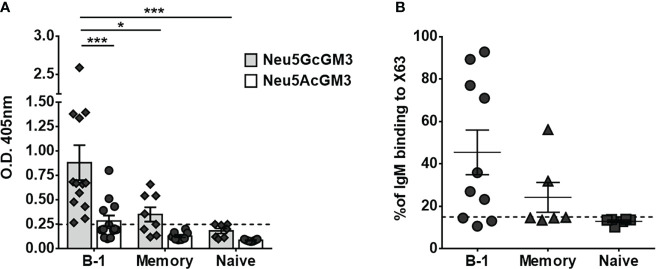
Secretion of anti-Neu5GcGM3 IgM by different B-cell subsets. Sort purified B-cell subsets were stimulated *in vitro* with CpG, anti-Igs, and IL-2. Cell-culture supernatants were collected after 7 days and analyzed for IgM reactive to Neu5GcGM3 and Neu5AcGM3 gangliosides by ELISA **(A)** or for IgM binding to Neu5GcGM3+ X63 tumor cell line by flow cytometry **(B)**. **(A)** Bars represent media±SEM, and dots denote values obtained for each individual healthy donor. Dotted line represents the minimum absorbance value (0.25) from which a sample was considered positive. P values were calculated by two-way ANOVA with Sidak’s multiple comparisons test. **(B)** Cells incubated only with FITC-conjugated anti-human IgM were the negative control. Each dot denotes values obtained for an individual healthy donor and data shown are representative of two experiments performed. Dotted line represents the positive criteria: the percentage of binding ≥15% and at least two times the percentage obtained for the negative control *p < 0.05; ***p < 0.001.

To identify the human B-cell subset that naturally secretes anti-Neu5GcGM3 antibodies, we stimulated purified B-cell populations for 7 days. Cell culture supernatants from mature/naive, memory and B-1 cells were screened by ELISA against Neu5GcGM3 and its acetylated variant Neu5AcGM3, used as a specificity control. As shown in **Figure 2A**, all the B-1 cell supernatants from 13 tested donors had IgM reactive to Neu5GcGM3 (100% of samples). Of those, 4 B-1 samples had also IgM antibodies cross-reactive with Neu5AcGM3 at a lower level (**Figure 2A**). In contrast, no mature/naive B-cell supernatant had IgM antibodies specific for any of the gangliosides and only 4 out of 8 supernatants (50%) from memory cells reacted against Neu5GcGM3. No anti-Neu5AcGM3 antibodies were detected in mature/naive or memory subsets.

Next, we evaluated whether the anti-Neu5GcGM3 antibodies present in B-cell culture supernatants were able to recognize the ganglioside exposed on the cytoplasmic membrane of tumor cells. The supernatants were incubated with the murine myeloma X63, which expresses high levels of Neu5GcGM3 ganglioside (23) and the IgM binding to cells was measured by flow cytometry. As shown in **Figure 2B**, seven out of ten B-1 cell culture supernatants and two out of four memory B-cell supernatants that showed reactivity against Neu5GcGM3 by ELISA also recognized X63 tumor cells. In line with the ELISA results, neither of the mature/naive nor the two memory supernatants that were negative by ELISA, recognized the tumor cells. These results corroborate the ability of human B-1 cells to participate in the immune surveillance against tumors expressing Neu5GcGM3.

## Discussion

NAbs play a fundamental role in the defense against infectious pathogens (24–26) and in the elimination of noxious structures like dead cells and oxidized epitopes (27, 28). Some NAbs are also able to eliminate tumor cells (19, 20, 29), pointing to a relevant role for these immunoglobulins and the B-1 cells secreting them in the defense against malignant transformations. Neu5GcGM3 ganglioside is not only overexpressed in several human tumors, but is also relevant for tumor biology (30) and exerts an immunosuppressive effect that promotes tumor growth (31, 32). We have previously detected anti-Neu5GcGM3 antibodies in healthy humans. These antibodies recognize and eliminate tumor cells expressing Neu5GcGM3 ganglioside (3). Due to these properties, naturally-occurring anti-Neu5GcGM3 antibodies may be crucial as a first line defense against tumors, not only by eliminating transformed cells but also by inhibiting the immunosuppressive effect of Neu5GcGM3. Interestingly, the level of naturally-occurring anti-Neu5GcGM3 antibodies significantly decreases in the elderly, when cancer incidence is higher (3). Similarly, an age-associated decline in naturally-occurring antibodies levels against other disease-associated epitopes have been reported (33, 34). These results support the idea that the decrease or absence of homeostatic NAbs increases the risk of developing certain diseases whose incidence rises in the older age population (2).

Possible explanations for the decrease in protective antibody levels with increasing age involve a reduction in the frequency, an impaired function, or a shift in the repertoire of the B-cell population secreting these antibodies. We previously showed that murine B-1 cells contain on their repertoire anti-Neu5GcGM3 antibodies able to bind and eliminate Neu5GcGM3-positive tumor cells (21). In 2011, Griffin et al. described a human B-cell population equivalent to mouse B-1 cells (9), that was recently validated in the prenatal scenario (16). This population has been shown to secrete antibodies that share the same binding specificities than those secreted by murine B-1 cells, like the recognition of PC and oxLDL (10, 35). However, human B-1 cells have not yet been tested for production of antibodies against Neu5GcGM3.

Here we confirm our previous observation on the existence of anti-Neu5GcGM3 antibodies in healthy humans (3). We detected IgM antibodies reactive to this ganglioside in 46% of the plasma samples that were analyzed. The donors with circulating anti-Neu5GcGM3 IgM antibodies had an increased percentage of B-1 cells in comparison with those who do not have anti-Neu5GcGM3 antibodies. In contrast, the frequency of mature/naive was lower in the healthy donors with a positive response to Neu5GcGM3, and the memory B-cell percentage was the same, independently of the presence of circulating anti-Neu5GcGM3 antibodies. These results suggested that human B-1 cells may be involved in the secretion of anti-Neu5GcGM3 IgMs in healthy donors. A similar correlation was established by Fiskesund et al. for anti-PC antibodies (35). According to these authors, the relative number of PC-reactive CD27+CD43+ B cells identified by flow cytometry in healthy donor’s peripheral blood correlates significantly to the serum level of anti-PC IgM, suggesting that these cells are a fundamental antibody-secreting population.

In an attempt to identify, for the first time, which human B-cell subtype was responsible for anti-Neu5GcGM3 IgM secretion, we cultured different B-cell populations with a polyclonal stimulation that assured immunoglobulin secretion and the supernatants were tested against Neu5GcGM3 by ELISA. Among those tested human B-1, memory, and mature/naive B cell subsets, IgM anti-Neu5GcGM3 antibodies were generated predominantly by human B-1 cells. Although we were able to induce general IgM secretion in all B-cell subtypes, we did not detect any specific binding to Neu5GcGM3 in the cell culture supernatant of mature/naive cells. However, we did detect anti-Neu5GcGM3 antibodies in some memory samples. It is possible that the Neu5GcGM3 reactivity is present at very low levels in the naive repertoire and becomes detectable in the memory repertoire after the selection an amplification induced by an antigenic encounter. Nevertheless, even though both memory and B-1 cells secrete anti-Neu5GcGM3 antibodies, the frequency of this specificity, as well as the intensity of the response, were higher in the B-1 repertoire. These results suggest human B-1 cells are an important contributor to circulating IgM anti-Neu5GcGM3 antibodies, and probably have an immune surveillance function associated with this reactivity. In fact, most of B-1 cell samples recognizing Neu5GcGM3 by ELISA also bound to this antigen in its natural conformation on tumor cell membranes.

The relevance of anti-Neu5GcGM3 antibodies in cancer treatment is currently studied in clinical trials. Cancer patients vaccinated with the anti-idiotypic antibody racotumomab (that displays the “internal image” of Neu5GcGM3) developed not only IgM but also IgG anti-Neu5GcGM3 antibodies (36–39). Racotumomab has been used as a switch maintenance therapy in advanced NSCLC patients. In this clinical trial, racotumomab prolonged overall survival and progression-free survival. Interestingly, patients that developed anti-Neu5GcGM3 IgM antibodies able to bind and eliminate NeuGcGM3-expressing tumor cells had the best outcomes (40). Thus, either natural or induced cytotoxic anti-Neu5GcGM3 IgM antibodies appear to be protective against tumors expressing this antigen. We are working on the detection of Neu5GcGM3-specific B cells in NSCLC patients treated with racotumomab, to assess the use of these B cells as prognostic factors of the disease outcome and to evaluate their correlation with the response to treatment.

Human B-1 cells level significantly declines with age (9, 41). Furthermore, the ability of human B-1 cells to spontaneously secrete IgM antibodies is adversely affected by aging (41). Here, we identified human B-1 cells as the main population responsible for natural anti-Neu5GcGM3 IgM secretion in healthy individuals. Consequently, a decline on B-1 cell number and function may explain the decrease on naturally-occurring anti-Neu5GcGM3 antibodies in healthy individuals during aging. Beside a cell intrinsic defect in spontaneous immunoglobulin secretion, there is also a reduction on the diversity of the B-1 cell IgM antibody repertoire from old donors (41). The disappearance of Neu5GcGM3 reactivity from the elderly B-1 cell repertoire could also explain the low levels of antibodies against this ganglioside detected in the old human population, a hypothesis that warrants future testing. The reduction of B-1 cells secreting antibodies with immune surveillance properties could contribute to the increased susceptibility of aged individuals to neoplastic transformation.

## Data availability statement

The original contributions presented in the study are included in the article/**Supplementary Material**. Further inquiries can be directed to the corresponding author.

## Ethics statement

The studies involving human participants were reviewed and approved by North Shore-LIJ Health System Institutional Review Board. The patients/participants provided their written informed consent to participate in this study.

## Author contributions

NRZ, TLR and AMH conceived the experiments. NRZ and TDQ carried out the experiments. NRZ analyzed data. TLR and AMH oversaw the project. All authors contributed to the article and approved the submitted version.

## Funding

This work was supported by the Center of Molecular Immunology and by U.S. Public Health Service grants AI 029690 and AI 142004 awarded by the National Institutes of Health.

## Acknowledgments

We thank the Clinical Research Service team for recruiting and collecting healthy volunteer’s samples. The authors thank all the volunteers who participated in this study.

## Conflict of interest

The authors declare that the research was conducted in the absence of any commercial or financial relationships that could be construed as a potential conflict of interest.

## Publisher’s note

All claims expressed in this article are solely those of the authors and do not necessarily represent those of their affiliated organizations, or those of the publisher, the editors and the reviewers. Any product that may be evaluated in this article, or claim that may be made by its manufacturer, is not guaranteed or endorsed by the publisher.

## Supplementary material

Supplementary Figure 1 Phenotypic analysis of human peripheral blood B-cell subsets by flow cytometry. Representative gating strategy to analyze and purify different human B-cell subsets. PBMCs were stained with Live/Dead Fixable Aqua Dead Cell Stain Kit and antibodies specific for human CD3, CD4, CD7, CD19, CD20, CD27, CD38, and CD43. After doublet and death cell discrimination, CD19+ cells were separated from CD3, CD4, CD7 positive cells. CD20+ cells were separate from CD20- plasma cells and plotted using CD27 and CD38 to exclude CD38^high^ pre-plasmablast. CD38^low/int^ were resolved according to CD27 and CD43 expression with mature/naive being CD27-CD43-, memory B cells CD27+CD43- and B-1 cell being CD27+CD43+. Fluorescence Minus One controls were used for CD27+ and CD43+ cells selection.
