# Supplementary figures and images for: Human B-1 cells are important contributors to the naturally-occurring IgM pool against the tumor-associated ganglioside Neu5GcGM3
Source: Front Immunol. 2022 Nov 30;13:1061651. doi: 10.3389/fimmu.2022.1061651 (PMC9747505; doi:10.3389/fimmu.2022.1061651)

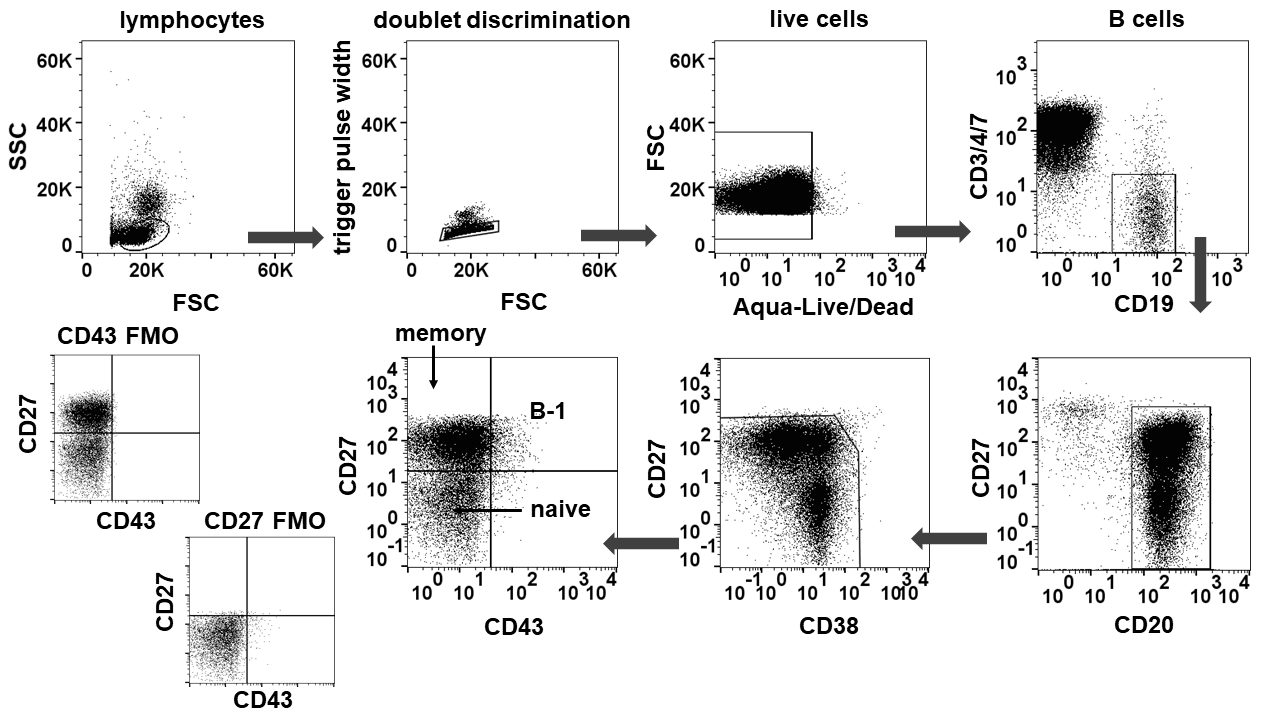

Supplement: Supplementary Figure 1 — Phenotypic analysis of human peripheral blood B-cell subsets by flow cytometry. Representative gating strategy to analyze and purify different human B-cell subsets. PBMCs were stained with Live/Dead Fixable Aqua Dead Cell Stain Kit and antibodies specific for human CD3, CD4, CD7, CD19, CD20, CD27, CD38, and CD43. After doublet and death cell discrimination, CD19+ cells were separated from CD3, CD4, CD7 positive cells. CD20+ cells were separate from CD20- plasma cells and plotted using CD27 and CD38 to exclude CD38high pre-plasmablast. CD38low/int were resolved according to CD27 and CD43 expression with mature/naive being CD27-CD43-, memory B cells CD27+CD43- and B-1 cell being CD27+CD43+. Fluorescence Minus One controls were used for CD27+ and CD43+ cells selection. [file Image_1.tif]
